# Selection and Validation of Suitable Reference Genes for RT-qPCR Normalization in *Euonymus bungeanus* Across Various Tissues and Under Abiotic Stress/Hormone Treatments

**DOI:** 10.3390/plants15081230

**Published:** 2026-04-16

**Authors:** Yongbin Ou, Hong Lu, Xincheng Zhao, Yueping Qin, Xinghong Zhong, Bo Zhou, Yinan Yao

**Affiliations:** College of Life Sciences and Agri-Forestry, Southwest University of Science and Technology, Mianyang 621010, China; lhongja@163.com (H.L.); xincheng01162022@163.com (X.Z.); 19381090985@163.com (Y.Q.); zxhswust@gmail.com (X.Z.); 15508053320@163.com (B.Z.)

**Keywords:** *Euonymus bungeanus*, reference gene selection, RT-qPCR normalization, abiotic stress, gene expression stability

## Abstract

*Euonymus bungeanus* is a highly valued ornamental tree/shrub species widely utilized in landscaping and afforestation in Northeast Asia, yet molecular studies on this species remain limited due to the lack of validated reference genes for reverse transcription quantitative real-time polymerase chain reaction (RT-qPCR). In this study, 16 candidate reference genes were selected based on classical plant reference genes and our previous transcriptome data. Their expression stability was comprehensively evaluated using 64 samples collected from diverse tissues and plants subjected to various abiotic stress/hormone treatments across multiple time points. Across all samples analyzed, *PBG1* (*20S proteasome beta subunit G1*) exhibited the highest overall expression stability, followed by *VAPD* (*vacuolar ATP synthase subunit D*) and *EIF4A* (*eukaryotic translation initiation factor 4A*). For tissue-specific analysis, *TSR2* (*pre-rRNA-processing protein*), *VAPD*, and *PBG1* demonstrated the greatest stability. Under specific stress conditions, *PBG1* and *EIF4A* were identified as the most stable genes under low- and high-temperature conditions. *PP2A* (*protein phosphatase 2A*) and *TUB6* (*beta-6 tubulin*) were optimal for drought stress, while *TSR2*, *SRP* (*nuclear speckle splicing regulatory-like protein*), and *PBG1* exhibited superior stability under salt stress. These findings establish a validated panel of reference genes enabling accurate and reliable gene expression normalization in *E. bungeanus*, thereby facilitating future functional genomics studies in this economically and ecologically important species.

## 1. Introduction

Winterberry euonymus (*Euonymus bungeanus* Maxim., also known as *Euonymus maackii* Rupr.), a deciduous shrub or small tree belonging to the family Celastraceae, is mainly distributed in regions north of the Yangtze River in China, as well as in southern Siberia and the Korean Peninsula. It is recognized as an excellent native tree species in northern China [1]. Its leaves turn a vibrant rose-red in autumn, rendering it a highly prized ornamental foliage plant. Flowering and fruiting typically occur 2–3 years after planting, with pink capsules and red arils further enhancing its ornamental value [2]. Owing to its rapid growth rate, tolerance to barren soils and pruning adaptability, this species has become increasingly prominent in urban landscaping and is also widely utilized as a street tree. Horticulturists have developed several improved cultivars, including ‘Golden Leaf’, ‘City of Flowers’ and ‘Golden Flame’ [3]. Additionally, *E. bungeanus* possesses a well-developed root system and strong stress resistance, making it suitable for afforestation in sandy and saline–alkaline soils of arid and coastal regions [4]. This species exhibits excellent cold tolerance but is sensitive to high temperatures. Several next-generation sequencing datasets for *E. bungeanus* and its close relatives have been deposited in public databases [5,6,7,8], and tissue culture and genetic transformation systems have been established [9]. Despite these advances, molecular studies on *E. bungeanus* remain relatively scarce.

Accurate analysis of gene expression patterns is a fundamental prerequisite for in-depth functional genomics research, including transcriptome validation and the identification and characterization of key genes involved in specific biological pathways. A variety of methodologies have been developed for gene expression analysis, among which RT-qPCR has emerged as a leading technique due to its simplicity, high sensitivity, specificity and reproducibility [10]. However, RT-qPCR assays are susceptible to variations arising from experimental protocols and technical inconsistencies, particularly in terms of RNA integrity, template loading quantity and reverse transcription efficiency [11]. Therefore, the selection of appropriate reference genes for data normalization is essential to ensure the accuracy and reliability of RT-qPCR results.

Reference genes are endogenous genes with stable expression across different tissues, developmental stages and environmental conditions [12]. Classic plant reference genes are predominantly housekeeping genes, including *ACT* (*actin*), *CYP5* (*cyclophilin 5*), *EF1α* (*elongation factor 1α*), *EIF4A* (*eukaryotic translation initiation factor 4A*), *GAPDH* (*glyceraldehyde-3-phosphate dehydrogenase*), *HIS3* (*histone superfamily protein*), *PP2A (protein phosphatase 2A*), *SAND* (*Sp100, AIRE-1, NucP41/75, DEAF-1 domain protein*), *TUB* (*tubulin*), *UBC9* (*ubiquitin-conjugating enzyme 9*), *UBQ* (*ubiquitin*) and *YLS8* (*mRNA splicing factor, thioredoxin-like U5 snRNP*) [13,14,15,16,17,18,19]. Nevertheless, the expression stability of these genes varies considerably among different plant species, and accumulating evidence indicates that even within the same species, no single reference gene maintains consistent stability across all tissue types and experimental conditions [12].

Careful selection and experimental validation of reference genes are crucial for robust RT-qPCR normalization. Several statistical algorithms have been developed to evaluate the expression stability of candidate reference genes, including GeNorm [20], NormFinder [21], BestKeeper [22], ∆Ct [23], and RefFinder [24]. These tools enable researchers to identify stably expressed reference genes under specific experimental conditions, thereby minimizing errors caused by inappropriate reference gene selection. In addition, advances in next-generation sequencing technologies have facilitated the use of omics data to identify novel candidate reference genes [12]. For instance, transcriptome data have been successfully employed to screen reference genes in *Arabidopsis thaliana* [17] and rice (*Oryza sativa*) [15,18], providing valuable insights for other species.

In this study, we selected 11 commonly used reference genes in model plants and five novel candidate genes from the transcriptome data of *E. bungeanus*. A total of 64 samples were collected, including diverse tissue types and leaf samples subjected to seven abiotic stress/hormone treatments: low temperature, high temperature, drought, sodium chloride (NaCl), hydrogen peroxide (H_2_O_2_), abscisic acid (ABA) and 1-aminocyclopropanecarboxylic acid (ACC) [25]. Following RT-qPCR analysis, the expression stability of the candidate reference genes was evaluated using GeNorm, NormFinder, BestKeeper, the ΔCt method and RefFinder to identify the most suitable reference genes for different experimental conditions. To further validate the reliability of the selected reference genes, two key genes involved in the ethylene biosynthesis pathway and leaf senescence [26,27], *ACS* (*ACC synthase*) and *ACO* (*ACC oxidase*), were used as target genes for expression analysis. This study systematically identifies the most stable reference genes for *E. bungeanus* across diverse tissues and experimental conditions, thereby laying a solid foundation for future research on the molecular mechanisms underlying stress resistance and functional genomics of this species.

## 2. Results

### 2.1. Selection of the Candidate Reference Genes

**Figure 1:**
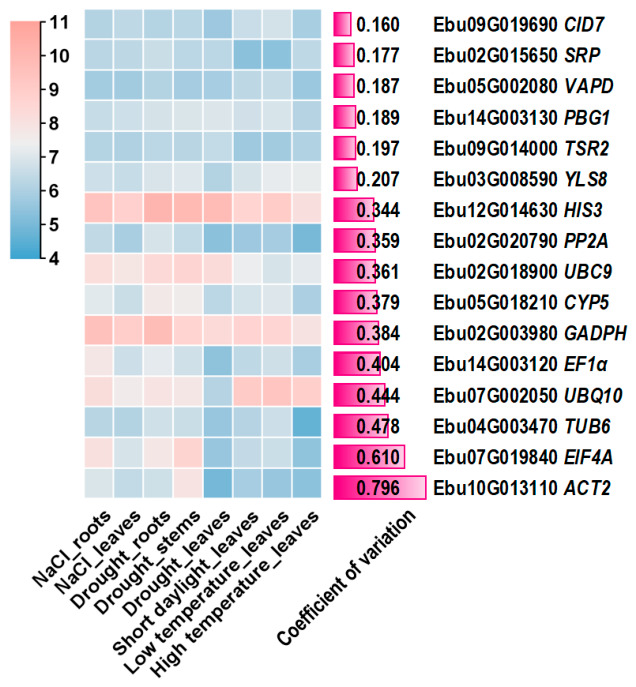
Expression patterns of candidate reference genes based on transcriptome data. The heatmap scale represents log_2_-transformed FPKM values; for example, a value of 6 corresponds to an expression level of 64 FPKM. The coefficient of variation (CV) was calculated using raw FPKM values rather than log_2_-transformed values.

We identified orthologous sequences of 11 candidate reference genes in *Euonymus bungeanus*, including *ACT*, *CYP5*, *EF1α*, *EIF4A*, *GAPDH*, *HIS3*, *PP2A*, *TUB*, *UBC9*, *UBQ*, and *YLS8*, and their expression profiles were examined using our previously generated transcriptome dataset comprising eight samples (Figure 1). Among these genes, *HIS3* exhibited the highest transcript abundance, with a maximum expression level of 1127 fragments per kilobase of exon model per million mapped fragments (FPKM) in drought-stressed roots and a mean expression level of 663 FPKM. Conversely, *PP2A* showed the lowest transcript accumulation, with a mean value of 66 FPKM. The coefficient of variation (CV) values of these 11 genes ranged from 0.207 to 0.796, indicating distinct expression variability. Despite their expression fluctuations, all genes were preserved for subsequent stability assessment owing to their extensive application as reference genes in plant studies.

From the same transcriptome dataset (containing 35,431 genes), 74 genes exhibited a CV below 0.200. Among these stably expressed genes, five genes including *CID7* (*CDC-interacting domain 7*), *SRP* (*nuclear speckle splicing regulatory-like protein*), *VAPD* (*vacuolar ATP synthase subunit D*), *PBG1* (*20S proteasome beta subunit G1*), and *TSR2* (*pre-rRNA-processing protein*), with mean expression levels exceeding 64 FPKM were selected as novel candidate reference genes (Figure 1).

### 2.2. Primer Specificity and Amplification Efficiency

**Figure 2:**
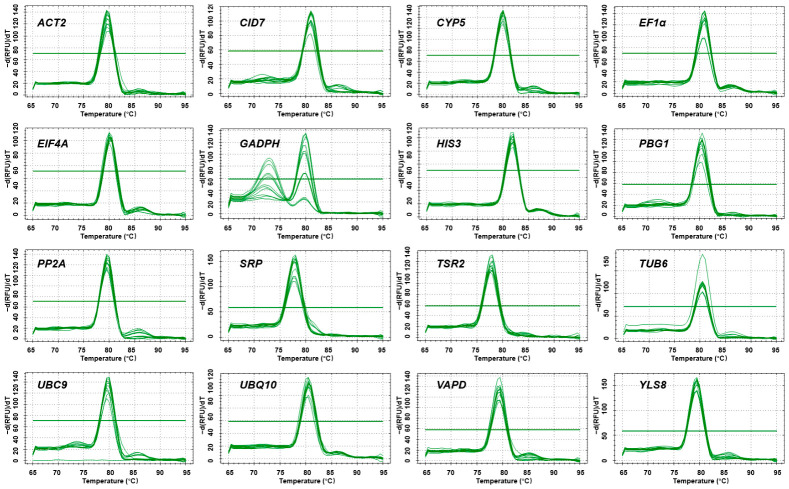
Melting curve analysis verifying the primer specificity of candidate reference genes, as visualized by Bio-Rad CFX Manager Software version 3.1.

The optimal amplification efficiency for RT-qPCR primers typically ranges from 90% to 110%. In the present study, three primer pairs (for *CID7*, *GAPDH* and *UBC9*) exhibited amplification efficiencies outside this range (Table 1). Melting curve analysis further revealed a double peak for *GAPDH*, with a non-specific peak at a melting temperature (Tm) of 72.7 °C when low-concentration templates were used, which was likely attributable to primer-dimer formation or non-specific amplification (Figure 2).

With the exception of *CID7*, *GAPDH* and *UBC9*, the remaining 13 primer pairs exhibited amplification efficiencies ranging from 91.21% (*YLS8*) to 101.83% (*UBQ10*), correlation coefficients (R^2^) from 0.983 (*SRP*) to 0.997 (*UBQ10*), and single specific peaks in melting curve analysis, confirming their high specificity and amplification efficiency. These 13 primer pairs were therefore considered suitable for subsequent RT-qPCR analysis. 

### 2.3. Distribution of Ct Values for Candidate Reference Genes

**Figure 3:**
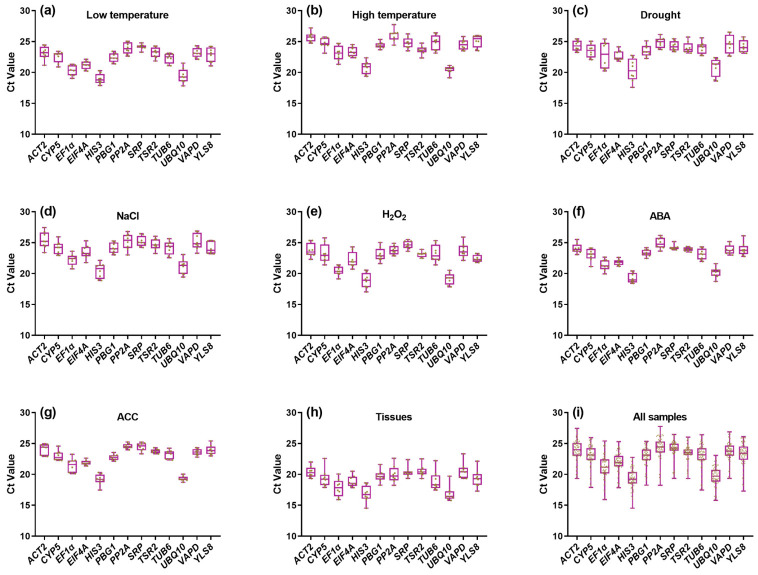
Box-plot visualization of Ct value distributions for 13 candidate reference genes across different sample sets. Ct value variability is shown for samples subjected to: (**a**) low temperature; (**b**) high temperature; (**c**) drought; (**d**) 600 mmol/L NaCl; (**e**) 1 mmol/L H_2_O_2_; (**f**) 0.1 mmol/L ABA; (**g**) 10 mmol/L ACC; (**h**) various tissues; and (**i**) all samples combined. The horizontal line within each box represents the median; box boundaries indicate the 25th and 75th percentiles; whiskers denote the maximum and minimum values.

When amplification efficiencies are comparable, the logarithm of gene expression abundance is inversely correlated with the threshold cycle (Ct) value. Among the candidate reference genes, *HIS3* exhibited the lowest Ct value (19.34 ± 1.63), whereas *PP2A* showed the highest Ct value (24.16 ± 1.94) (Figure 3), indicating that *HIS3* is highly expressed and *PP2A* is lowly expressed, which is consistent with transcriptome data (Figure 1). Notably, certain genes displayed condition-dependent expression patterns; for example, *ACT2* exhibited consistently higher Ct values under high-temperature or salt stress compared with other treatments.

In terms of Ct value distribution, *TSR2*, *SRP* and *PBG1* showed relatively narrow Ct ranges (6.70–7.10) and low CVs (0.058–0.067) (Figure 3i), suggesting stable expression across all samples. In contrast, *EF1α*, *TUB6* and *YLS8* displayed wider Ct ranges (8.84–9.47) and higher CVs (0.084–0.093), indicating greater expression variability. For individual stress/hormone treatments, the Ct values of most candidate genes remained relatively consistent across different time points (Figure 3a–g). However, across different tissue types, nearly all genes exhibited elevated Ct values in root samples (Figure 3h).

### 2.4. Expression Stability of Candidate Reference Genes Based on Four Statistical Algorithms

**Figure 4:**
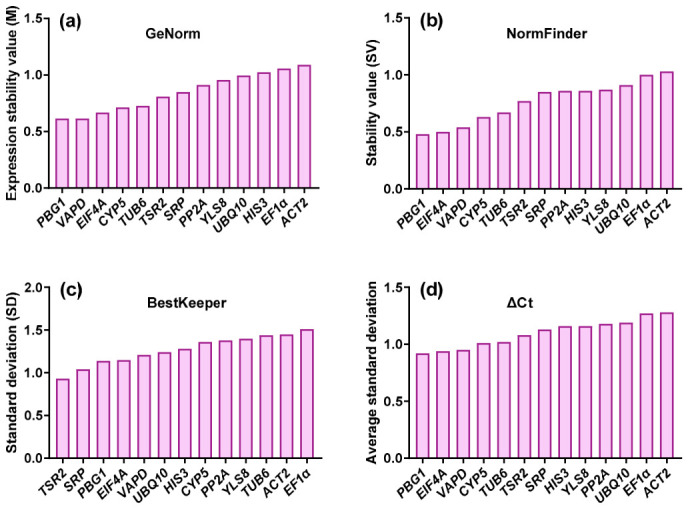
Expression stability of candidate reference genes in the combined sample set, as evaluated by four algorithms: (**a**) GeNorm, (**b**) NormFinder, (**c**) BestKeeper, and (**d**) the ΔCt method.

To evaluate the relative suitability of the 13 candidate reference genes, four commonly used statistical algorithms (GeNorm, NormFinder, BestKeeper and the ΔCt method) were employed (Figure 4).

GeNorm calculates pairwise variation and ranks genes based on the average expression stability value (M), with lower M values indicating higher expression stability. Analysis of the combined dataset of all samples revealed that all candidate genes exhibited M values below 1.5, meeting the criteria for valid reference genes. Among them, *PBG1* showed the highest stability, followed by *VAPD* and *EIF4A* (Figure 4a).

NormFinder evaluates both intra- and inter-group expression variation and generates a stability value (SV) for each gene, with lower SVs reflecting greater stability. *PBG1* had the lowest SV, followed by *EIF4A* and *VAPD* (Figure 4b).

BestKeeper ranks genes based on the standard deviation (SD) of Ct values, with lower SD indicating higher stability. *TSR2* displayed an SD below 1 (0.93), indicating strong expression stability, followed by *SRP*, and *PBG1*, *EIF4A* (Figure 4c).

The ΔCt method calculates the average SD of pairwise Ct value comparisons, with genes showing the lowest mean SD identified as the most stable. *PBG1*, *EIF4A* and *VAPD* emerged as the top three most stable genes (Figure 4d).

Overall, the rankings generated by the ΔCt method and NormFinder were largely consistent, while notable discrepancies were observed between these two algorithms and GeNorm/BestKeeper.

### 2.5. Comprehensive Stability Ranking Generated by RefFinder

**Figure 5:**
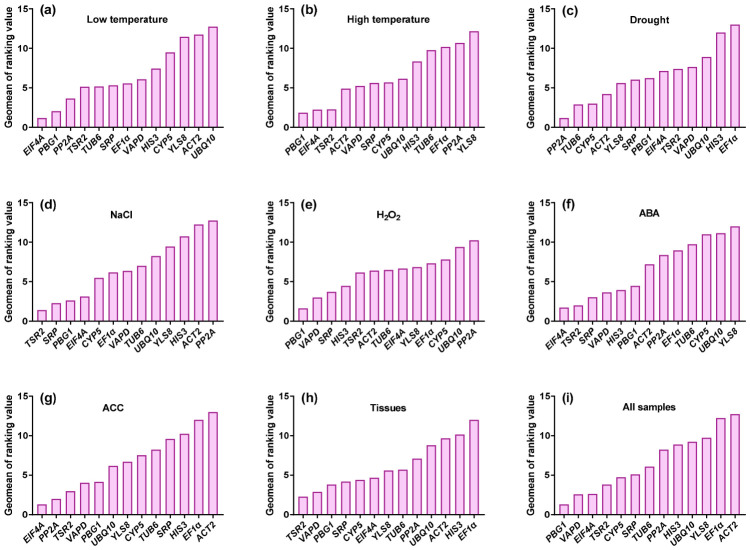
Comprehensive stability ranking of candidate reference genes generated by RefFinder using Ct values from the indicated sample sets: (**a**) low temperature; (**b**) high temperature; (**c**) drought; (**d**) 600 mmol/L NaCl; (**e**) 1 mmol/L H_2_O_2_; (**f**) 0.1 mmol/L ABA; (**g**) 10 mmol/L ACC; (**h**) various tissues; and (**i**) all samples combined. A higher geomean value indicates lower expression stability.

Given the variability in stability rankings among different algorithms, RefFinder was used to generate a comprehensive stability ranking by calculating the geometric mean of stability weights derived from GeNorm, NormFinder, BestKeeper and the ΔCt method (Figure 5). A higher geometric mean value corresponds to lower expression stability.

Across all samples, *PBG1* (geometric mean = 1.32) exhibited the highest overall expression stability, establishing it as the most reliable reference gene for normalizing gene expression across diverse tissues and experimental conditions in *E. bungeanus* (Figure 5i). *VAPD* (2.59) and *EIF4A* (2.63) also demonstrated strong stability and represent suitable alternatives for large-scale expression studies. In contrast, *EF1α* (12.24) and *ACT2* (12.47) were the least stable genes and are not recommended for RT-qPCR normalization in *E. bungeanus*.

Stability rankings varied across different treatment and tissue subsets (Figure 5a–h). Under low-temperature stress, *EIF4A* and *PBG1* were the most stable candidates (geometric mean < 3; Figure 5a). Under high-temperature stress, *PBG1*, *EIF4A* and *TSR2* showed the highest stability (Figure 5b). For drought stress, *PP2A*, *TUB6* and *CYP5* were the optimal reference genes (Figure 5c). Under salt stress, *TSR2*, *SRP* and *PBG1* ranked highest in stability (Figure 5d). For H_2_O_2_ treatment, *PBG1* and *VAPD* were the preferred reference genes (Figure 5e). Following ABA treatment, *EIF4A* and *TSR2* exhibited the greatest stability (Figure 5f). Under ACC treatment, *EIF4A*, *PP2A* and *TSR2* were the optimal candidates (Figure 5g). Across different tissue types, *TSR2*, *VAPD* and *PBG1* were the most stable genes (Figure 5h). Collectively, with the exception of drought stress, *PBG1*, *EIF4A* and *TSR2* consistently emerged as top candidates across most experimental conditions.

### 2.6. Optimal Number of Reference Genes for Normalization

**Figure 6:**
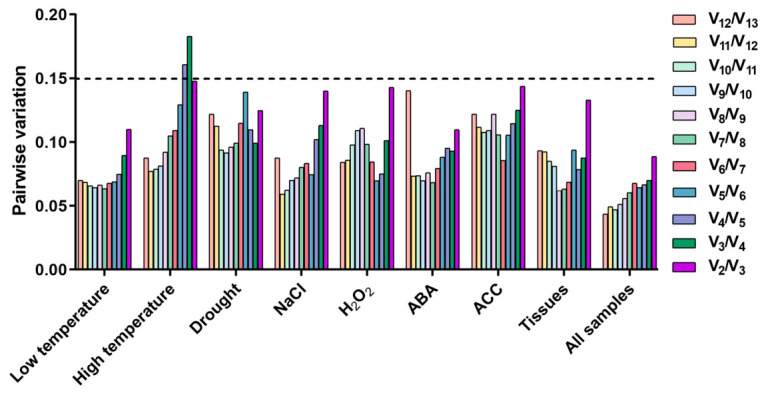
Pairwise variation (V*_n_*/V*_n_*_+ 1_) calculated by the GeNorm algorithm. The V*n*/V*_n_*_+ 1_ value indicates the improvement in expression stability when adding an (*n* + 1)th gene. The threshold for optimal reference gene number is set at 0.15.

The use of multiple reference genes can improve the accuracy of RT-qPCR data normalization. GeNorm calculates the pairwise variation (V*_n_*/V*_n_* _+ 1_) to determine the optimal number of reference genes, with a threshold of 0.15 indicating that the addition of more reference genes does not significantly improve normalization accuracy. Notably, V_2_/V_3_ pairwise variation values across all experimental conditions in this study were below 0.15 (Figure 6), suggesting that two stably expressed reference genes are sufficient for reliable RT-qPCR normalization in *E. bungeanus*.

### 2.7. Validation of Candidate Reference Genes

**Figure 7:**
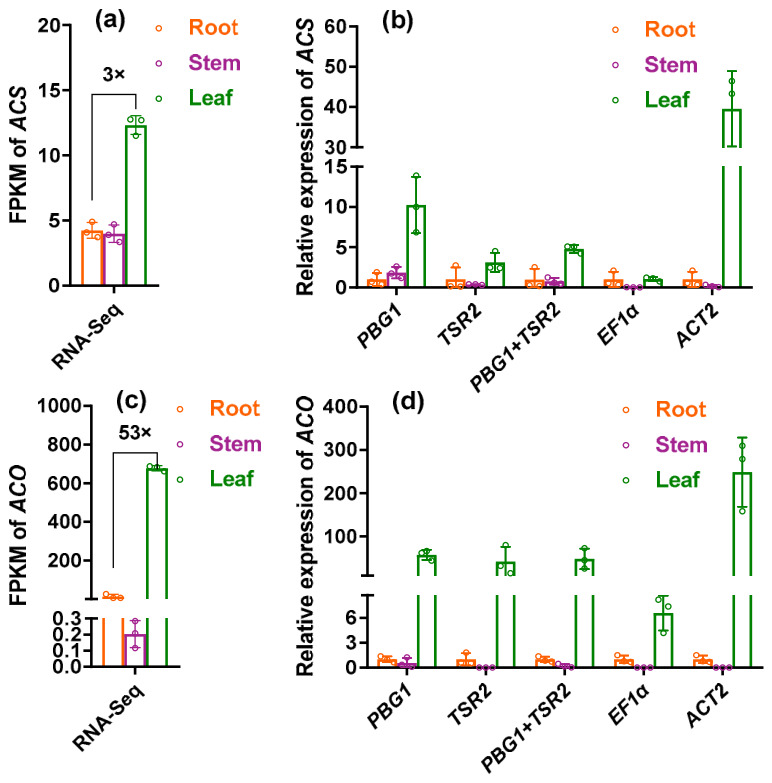
Relative expression of *ACS* and *ACO* in various tissues of *E. bungeanus*. (**a**) Expression profile of *ACS* based on RNA-seq data. (**b**) RT-qPCR analysis of *ACS* expression normalized using different reference genes. (**c**) Expression profile of *ACO* based on RNA-seq data. (**d**) RT-qPCR analysis of *ACO* expression normalized using different reference genes. For RNA-seq data, the numbers above the bars indicate the fold change in FPKM in leaves relative to roots. For RT-qPCR data, the expression level in roots was set to 1 for all reference genes used.

To validate the reliability of the selected reference genes, the expression levels of *ACS* and *ACO* in different *E. bungeanus* tissues were normalized using: (1) *PBG1* (the most stable reference gene across all samples); (2) *TSR2* (the most stable reference gene across tissue types); (3) a combination of *PBG1* and *TSR2*; and (4) and (5) the two least stable genes (*EF1α* and *ACT2*) as negative controls. Transcriptome data indicated that *ACS* expression in roots was 4.2 FPKM, with minimal expression differences between stems and roots, while leaf expression was approximately threefold higher than that in roots (Figure 7a). *ACO* expression in roots was 12.7 FPKM, 0.2 FPKM in stems, and 53-fold higher in leaves compared with roots (Figure 7c).

When normalized using the stable reference genes (*PBG1*, *TSR2* or their combination), the expression profiles of *ACS* and *ACO* closely matched the transcriptome data. For *ACS*, the relative expression in stems compared with roots was 1.8 (normalized with *PBG1*), 0.4 (*TSR2*), and 0.8 (*PBG1* + *TSR2*); the relative expression in leaves compared with roots was 10.3, 4.8, and 3.1, respectively (Figure 7b). For *ACO*, the relative expression in stems compared with roots was 0.5 (*PBG1*), 0.01 (*TSR2*), and 0.2 (*PBG1* + *TSR2*); the relative expression in leaves compared with roots was 57.2, 42.2, and 47.8, respectively (Figure 7d). In contrast, normalization with the unstable reference genes (*EF1α* or *ACT2*) produced severely distorted expression patterns. For example, the expression of both target genes in leaves was either underestimated (normalized with *EF1α*) or overestimated (normalized with *ACT2*), underscoring the risks of using inappropriate reference genes for RT-qPCR normalization. These findings confirm that *PBG1*, *TSR2* and their combination are suitable for accurate gene expression normalization in *E. bungeanus*.

## 3. Discussion

RT-qPCR is one of the most widely used techniques for gene expression analysis in molecular biology; however, numerous factors such as RNA integrity, template loading quantity and reverse transcription efficiency can affect the accuracy, consistency and reproducibility of experimental results in practical applications [11]. Among these factors, the careful selection and experimental validation of reference genes are critical to ensure their suitability for specific tissue types, developmental stages and environmental conditions [12]. In this study, we selected 11 traditional plant reference genes and five novel candidate genes to identify optimal reference genes for RT-qPCR normalization in *E. bungeanus*, ultimately retaining 13 genes for subsequent stability analysis.

### 3.1. Performance of Traditional Reference Genes Under Diverse Experimental Conditions

Among the traditional reference genes assessed, *EIF4A* was identified as the optimal reference gene under low-temperature, ABA, and ACC treatment conditions, while *PP2A* was the most suitable under drought stress. In contrast, *UBQ10*, *ACT2*, and *EF1α* were found to be unsuitable as reference genes in *E. bungeanus*. Notably, *EIF4A* was also reported as the most stable reference gene during callus induction and redifferentiation in 84K poplar (*Populus alba × Populus glandulosa*), but exhibited relatively poor expression stability across different tissues at various developmental stages; conversely, *PP2A* was the most stable reference gene across poplar tissues of different ages, yet showed poor stability during callus induction and redifferentiation [16]. In tea plant (*Camellia sinensis*), *EIF4A* displayed low stability across different leaf developmental stages and under treatments with phytohormones such as gibberellins, 3-indoleacetic acid, salicylic acid, methyl jasmonate, and ABA [28], while *PP2A* exhibited the highest stability under heavy metal stress including manganese, aluminum, copper, iron, and zinc [29]. Consistent with our findings, *EF1α* was reported to be unstable across various tissues and organs at different developmental stages in tomato (*Solanum lycopersicum*) [30], but highly stable in rice (*Oryza sativa*) tissues [31]. In *Populus euphratica*, the optimal reference gene varied depending on the treatment condition, with *EF1α* being a suitable choice under low-temperature stress [32]. These studies clearly demonstrate that no single reference gene is universal for all plant species and experimental conditions. When performing gene expression analysis in a new species, orthologs of classical plant reference genes cannot be directly applied for RT-qPCR normalization without experimental validation.

### 3.2. Characterization of PBG1 and TSR2 as Novel Reference Gene

*PBG1* was identified as a broadly applicable reference gene in *E. bungeanus*, exhibiting stable expression across various experimental conditions, including low temperature, high temperature, salt stress, H_2_O_2_ treatment, different tissue types, and the combined samples. To our knowledge, this is the first report of *PBG1* being utilized as a reference gene for RT-qPCR in plants. *PBG1* encodes a 236-amino-acid protein that shares 91% sequence similarity with *Arabidopsis thaliana* AtPBG1 [33] (AT1G56450), 70% similarity with *Homo sapiens* HsPSMB4 [34] (Genpept accession: P28070), and 63% similarity with *Saccharomyces cerevisiae* ScPRE4 [35] (Genpept accession: P30657). PBG1 is a core subunit of the 20S proteasome and is highly conserved in eukaryotes, containing a conserved “proteasome_beta_type_4” domain spanning approximately 200 amino acids [36]. The 20S proteasome serves as the core enzyme for non-lysosomal protein degradation in both the cytosol and nucleus of eukaryotic cells. It is composed of 28 subunits arranged as four stacked homoheptameric rings, forming an elongated α-β-β-α cylinder with a central cavity. The 20S proteasome maintains cellular protein homeostasis by specifically degrading target proteins, either independently or as part of the ubiquitin–proteasome system. Typically, target proteins are ubiquitinated and subsequently recognized by the 19S regulatory subunit of the 26S proteasome, followed by degradation by the 20S core subunit [37]. However, it was reported that certain intrinsically disordered proteins (IDPs) can be directly degraded by the 20S proteasome without prior ubiquitination [38]. IDPs lack a fixed three-dimensional structure (either completely or partially), and their unstructured amino acid sequences render them susceptible to direct degradation by the 20S proteasome. Given that PBG1, together with the products of the classical reference genes *UBC* and *UBQ*, is a component of the cellular protein degradation system, it may possess great potential as a reference gene across multiple plant species, although further experimental validation is required.

*TSR2* is another novel gene identified as a suitable reference gene in *E. bungeanus*, exhibiting the highest expression stability under salt stress (across different time points) and across diverse tissue types. *TSR2* encodes a 208-amino-acid protein that shares 60% sequence similarity with *Arabidopsis thaliana* AtTSR2 (AT5G27990) and 50% similarity with *Saccharomyces cerevisiae* ScTSR2 (Genpept accession: Q06672), with sequence conservation restricted to a conserved WGG motif of approximately 100 amino acids [36]. TSR2 is involved in the maturation of large subunit (LSU)-rRNA and small subunit (SSU)-rRNA in eukaryotes, and mutations in the human ortholog of this gene have been implicated in Diamond-Blackfan anemia 14 with mandibulofacial dysostosis [39].

### 3.3. Challenges and Prospects for Reference Gene Selection in Plants

In the present study, the stability rankings of reference genes obtained from different datasets were inconsistent. Selecting appropriate reference genes that are suitable for as many different tissues, developmental stages, and various types of stress as possible remains a major challenge. Notably, with the exception of drought stress, most of the four novel candidate reference genes evaluated in this study exhibited expression stability ranked within the top 6 (top 50%) under most conditions. Exceptions included *VAPD* under low temperature or salt stress and *SRP* under ACC treatment. These results indicate that it is highly feasible to identify superior reference genes from transcriptome data. Although numerous transcriptome datasets are deposited in public databases, differences in sequencing methods and other factors necessitate the use of tools such as RefGenes [40] and RGeasy [41] to improve the reproducibility of reference gene selection through dataset integration [42]. With the development of high-throughput sequencing and multi-omics technologies, innovative methods for reference gene selection continue to emerge, providing higher precision for plant gene expression research.

## 4. Materials and Methods

### 4.1. Plant Materials and Treatments

One-year-old *Euonymus bungeanus* (genotype WM-7) seedlings were used as plant materials and grown in plastic pots (20 cm in diameter and 20 cm in height) filled with a 1:1 (*v*/*v*) mixture of garden soil and humus soil. The seedlings were cultivated in a glass greenhouse at Southwest University of Science and Technology (31°32′5′′ N, 104°42′0′′ E) under natural light conditions and regularly watered to maintain soil moisture. After two months of normal growth (regular irrigation, timely weeding, and targeted pest control), seedlings with consistent height, ground diameter and growth status were selected for experimental treatments and sample collection. A total of 64 seedlings were selected and divided into eight groups, with nine seedlings per group, and every three seedlings constituted a biological replicate.

For tissue-specific sampling, young leaves, mature leaves, petioles, young stems, mature stems, bark, buds and roots were collected separately, with three biological replicates for each tissue type.

Seven experimental treatments (low temperature, high temperature, drought, salt stress, H_2_O_2_, ABA and ACC) were conducted, all initiated between 9:00 a.m. and 10:00 a.m. Leaf samples were collected at 0 h, 2 h, 4 h, 8 h, 12 h, 16 h, 20 h and 24 h after the initiation of each treatment; leaves at the same position on the seedlings were collected at each time point, with three biological replicates per time point. All collected samples were immediately wrapped in aluminum foil, rapidly frozen in liquid nitrogen, and stored at −80 °C for subsequent RNA extraction and RT-qPCR analysis.

For low- and high-temperature treatments, three plant growth incubators were set to 28 °C (control), 4 °C (low temperature) and 42 °C (high temperature), respectively. Seedlings were acclimated in the 28 °C incubator for 2 days and then transferred to the 4 °C or 42 °C incubators; the 0 h sample was collected immediately upon transfer.

For drought treatment, seedlings were carefully removed from pots, and most of the soil adhering to the roots was removed to simulate natural drought conditions; the 0 h sample was collected immediately after soil removal.

For salt stress treatment, seedlings in pots were placed in plastic trays. After collecting the 0 h sample, 1 L of 600 mmol/L NaCl solution was poured into each pot. One hour later, the trays were removed, and any leachate in the trays was poured back into the corresponding pots; no further leachate was collected thereafter.

For H_2_O_2_, ABA and ACC treatments, aqueous solutions of the respective reagents were prepared as follows: 1 mmol/L H_2_O_2_ was prepared by pipetting 20.4 μL of 30% (*v*/*v*) H_2_O_2_ stock solution and diluting to 200 mL with distilled water (freshly prepared before use); 0.1 mmol/L ABA and 10 mmol/L ACC were prepared by dissolving 5.29 mg of ABA powder and 202.2 mg of ACC powder (weighed using an analytical balance with 0.0001 g precision) in 200 mL of distilled water, respectively. The prepared solutions were uniformly sprayed onto the leaves of *E. bungeanus* seedlings using a sprayer until slight dripping from the leaves was observed.

### 4.2. RNA Extraction and cDNA Synthesis

Total RNA was extracted from all samples using the OmoniPlant RNA Kit (CWBIO, Taizhou, China). Prior to extraction, mortars of appropriate size were cleaned and sterilized by baking at 180 °C for 2 h, then cooled to room temperature. Plant samples were ground into a fine powder in liquid nitrogen, and total RNA was extracted following the manufacturer’s instructions. Strict precautions were taken throughout the process to prevent RNA degradation. The extracted total RNA was fully dissolved in DEPC-treated water containing RNaseOff RNase Inhibitor (CWBIO, Taizhou, China). RNA integrity was assessed by 1% agarose gel electrophoresis, and RNA concentration and purity were determined using a microvolume spectrophotometer (Quawell, San Jose, CA, USA).

For cDNA synthesis, precisely 1 μg of high-quality total RNA (OD_260_/OD_280_ = 1.8–2.1) from each sample was used as the template. Reverse transcription was performed using the HiFiScript All-in-one RT Master Mix for qPCR (CWBIO, Taizhou, China) following the manufacturer’s recommended reaction system and amplification program. The resulting cDNA was diluted with ddH_2_O to a concentration corresponding to 5 ng of the original total RNA per μL and stored at −20 °C for subsequent RT-qPCR analysis.

### 4.3. Reference Gene Selection and Primer Design

Reference genes commonly used for RT-qPCR in model plants (*Arabidopsis thaliana*, *Oryza sativa*, *Populus trichocarpa*) were retrieved from the literature and public databases. Their orthologous sequences in *E. bungeanus* were identified via local BLAST searches embedded in TBtools v2.441 [43] and phylogenetic tree construction [44] based on our previously obtained genomic data (PRJNA1248448). By integrating transcriptome data (PRJNA1248448) from different tissues (roots, stems, leaves) and various stress treatments (45 °C for 6 h, 4 °C for 1 d, natural drought for 5 d, high salt for 24 h) of *E. bungeanus*, 11 candidate reference genes were selected from these classical reference genes (Figure 1). Additionally, five novel candidate reference genes were selected based on the criteria of a mean expression level greater than 64 FPKM and a CV less than 0.200 from the transcriptome dataset.

Specific PCR primers for all candidate reference genes were designed using NCBI Primer-BLAST [45] (https://www.ncbi.nlm.nih.gov/tools/primer-blast/, accessed on 12 March 2025) (Table 1). The primer design followed the following principles: amplicon length of 100–250 bp; Tm value of 60 ± 1 °C; primer length of 20 ± 1 bp. In addition, specific qPCR primers were also designed for two key genes in the ethylene biosynthesis pathway (*ACS* and *ACO*) to validate the reliability of the selected reference genes.

### 4.4. Quantitative PCR Conditions

Real-time quantitative PCR was performed on a CFX96 Real-Time PCR System (Bio-Rad, Hercules, CA, USA). The 20 μL PCR reaction system contained 10 μL of SuperStar Universal SYBR Master Mix (CWBIO, Taizhou, China), 2 μL of forward primer (3 μmol/L), 2 μL of reverse primer (3 μmol/L), 2 μL of cDNA template and 4 μL of ddH_2_O. For all regular RT-qPCR reactions (excluding primer efficiency analysis), the cDNA template amount corresponded to 10 ng of the original total RNA. To determine primer amplification efficiency and specificity, cDNA from different tissue types was pooled, and four serial 4-fold dilutions of the pooled cDNA were prepared and used as templates for RT-qPCR; amplicon dissociation curves were generated to assess primer specificity. The PCR reaction program was performed according to the manufacturer’s instructions, with the annealing temperature adjusted based on the predicted Tm values of the primers. All RT-qPCR analyses comprised three biological replicates and three technical replicates (three identical PCR amplifications).

### 4.5. Experimental Data Analysis and Processing

Ct values for each amplification reaction were calculated using Bio-Rad CFX Manag-er software version 3.1 (Bio-Rad, Hercules, CA, USA). The expression stability of the candidate reference genes was evaluated using five statistical tools: GeNorm [20], NormFinder [21], BestKeeper [22], the ΔCt method [23], and RefFinder [24], with default parameters applied for all analyses. For primer amplification efficiency analysis, standard curves were generated using the serial 4-fold cDNA dilutions, and the slope (k), correlation coefficient (R^2^) and amplification efficiency (E = (10^−1/k^ − 1) × 100%) were calculated using Bio-Rad CFX Manager software version 3.1. The relative expression levels of the target genes (*ACS* and *ACO*) were calculated according to the Livak method (2^−ΔΔCt^) [46] using the same software.

## 5. Conclusions

To facilitate future gene expression studies in *Euonymus bungeanus*, this study systematically evaluated 11 traditional plant reference genes and five novel candidate genes for RT-qPCR normalization, with 13 genes passing primer specificity and amplification efficiency validation. *PBG1* was identified as a broadly applicable reference gene, exhibiting stable expression across various experimental conditions, including low temperature, high temperature, salt stress, H_2_O_2_ treatment, and different tissue types. *EIF4A* emerged as the most suitable reference gene under low-temperature, ABA, and ACC treatments. *TSR2* demonstrated the highest expression stability under salt stress and across diverse tissue parts, while *PP2A* was the optimal reference gene for drought stress conditions. Based on these findings, we recommend the selection of a combination of two reference genes from *PBG1*, *TSR2*, *EIF4A* and *PP2A* according to specific experimental designs for accurate RT-qPCR normalization in *E. bungeanus*. These results lay a solid foundation for future functional genomics research on this economically and ecologically important ornamental tree species.

## Figures and Tables

**Table 1:** Amplification characteristics of the primers used in this study.

Gene Symbol	Arabidopsis Homolog	Gene Description	Primer Sequence (5′-3′)	Annealing Temperature (°C)	Amplicon Length (bp)	Amplification Efficiency (%)	R^2^
*ACT2*	AT3G18780	Actin 2	F: TACGAACTGCCTGATGGACAG R: CTGGGGGCAAGAGCAGTTATT	59.8	251	96.92	0.996
*CID7*	AT2G26280	CDC-interacting domain 7	F: CATTGGCAACAAAGCCCTAGC R: CCTGTAAACGCTTATCTGCTGC	62.4	263	84.46	0.990
*CYP5*	AT2G29960	Cyclophilin 5	F: CGATGGACGAGGTGGAGAATC R: CTTGGGTTGCCCATTCTGTTG	61.9	250	93.11	0.994
*EF1α*	AT5G60390	Elongation factor Tu family protein	F: GCTTCACCTCTCAGGTCATCA R: GCATGTCCCTCACAGCAAAAC	58.8	273	97.73	0.984
*EIF4A*	AT3G13920	Eukaryotic translation initiation factor 4A 1	F: CTGGGTCATCTCGCGTTCTAA R: AACATTTGCAGGCAGCTCTTC	60.0	248	93.23	0.996
*GADPH*	AT1G13440	Glyceraldehyde-3-phosphate dehydrogenase C2	F: TTCCCACTGTTGATGTCTCGG R: ATACTTGACCTGCTGTCACCG	60.5	178	327.01	0.909
*HIS3*	AT4G40030	Histone superfamily protein	F: GTTGCCCTTCGTGAAATTCGG R: CGTTCACCCCTGATCCTCCTA	62.8	266	98.40	0.996
*PBG1*	AT1G56450	20S proteasome beta subunit G1	F: TGGTGTCCATGATAGGTGTGC R: CCCTTGCTCTGTGATCTTTGC	59.2	212	100.85	0.990
*PP2A*	AT1G13320	Protein phosphatase 2A subunit A3	F: GCAATCCAGCATATTCTTCCCTG R: AAGCCGAACTCTCCAGTGTC	59.6	300	92.75	0.991
*SRP*	AT2G27285	Nuclear speckle splicing regulatory-like protein	F: TGAAGGTGAAACATCGTCCGT R: CATTTGTTGATCCGCACCAGG	61.8	127	96.05	0.983
*TSR2*	AT5G27990	Pre-rRNA-processing protein	F: CATCAGCATATAAGACAGGCGG R: AGTTGTCTCCTTGAACTTGGCT	58.8	168	98.28	0.987
*TUB6*	AT5G12250	Beta-6 tubulin	F: TTGTGATATCCCACCTAAGGGC R: GGCATCCTGGTATTGCTGGTA	60.4	226	94.41	0.996
*UBC9*	AT4G27960	Ubiquitin-conjugating enzyme 9	F: CAGGTGGAGTTTTCCTGGTGA R: GTCATCAGGATTTGGGTCCGT	59.7	215	122.58	0.986
*UBQ10*	AT4G05320	Polyubiquitin 10	F: GAGTCTACGCTGCATCTGGTG R: GCTTCCCTGCAAAGATCAACC	59.7	184	101.83	0.997
*VAPD*	AT3G58730	Vacuolar ATP synthase subunit D	F: GCTTGCTTCACTGCAGACATC R: CTTCTCCTCAGCAAACCCCTT	59.2	241	99.85	0.986
*YLS8*	AT5G08290	mRNA splicing factor, thioredoxin-like U5 snRNP	F: TACTGGCATCAGTGGCTGAAA R: CTTTTGGAGCGATTACCAGGC	59.7	273	91.21	0.988
*ACS*	AT1G62960	ACC synthase 10	F: CTATTTCAGCCCCAACCCAAC R: GCTCAAACTCCCCCTTCTCAC	59.2	228	99.18	0.993
*ACO*	AT1G62380	ACC oxidase 2	F: CCTCAAAGACGGCGAATGGAT R: GTTTCTCCTCCTCCTCTTCTCC	59.2	239	98.56	0.992

Note: The last two genes are target genes for validation of the candidate reference genes; R^2^: The linear regression correlation coefficients in RT-qPCR analysis.

## Data Availability

The data presented in this study are available upon request from the corresponding author.
